# Commentary - Radiology in India: The Next Decade

**DOI:** 10.4103/0971-3026.41867

**Published:** 2008-08

**Authors:** Dhandhapany Ragavan

**Affiliations:** Executive Vice President, Siemens Medical Solutions, 130 PB Marg, Worli, Mumbai - 400 018, India. E-mail: sujoymani@gmail.com

To understand the outlook for radiology, let us look at three major factors that will impact radiology and radiologists:

1. Growth potential and structural changes in the health care delivery system
2. Technological trends
3. Patient awareness/expectations and regulatory and quality trends.

## Growth Potential

The Indian health care infrastructure is grossly underdeveloped. Here is a quick look at some data pertaining to radiology (see Table 1).

**Table 1:** Comparision of CT and MRI scanners in different countries

Modality	Total installed base in India	(per million population) India	(per million population) China	(per million population) Japan (data as per OECD publications)	(per million population) Korea (data as per OECD publications)
CT	Approx. 300	3	Approx. 5.5	92.6	31.9
MRI	Approx. 600	0.6	Approx. 2	>35	>11

The commonest explanation offered for such a low installed base is that the affordability of Indian patients is significantly lower than that of patients in many other countries. However, I am convinced that affordability is just one part of the problem. The other two equally important issues are a) accessibility and b) awareness. Accessibility to good-quality health care is highly skewed in our country. For example, it is possible to find over 10 MRI scanners within a radius of 5 km in the city of Delhi, whereas in the entire State of Bihar the total number of installed MRI scanners would be around 10!! I present the example of Bihar only to convey the point clearly. The low numbers in Bihar or UP are not entirely due to a low paying capacity, but also due to the fact that health care facilities are not well distributed and awareness about the clinical role of these technologies is rather low. This is true for most of the country. However, we are now seeing positive changes: health care facilities are now spreading across the country and this will definitely spur the growth of radiology further.

## Structural Issues

Over 80% of secondary and tertiary care is delivered by the private sector, while the rest is delivered by the government sector. While this ratio is likely to remain the same in the foreseeable future, within the private sector, the structural changes that are underway are significant. On the one hand, private health care is getting more and more corporatized and on the other, there is a clear trend to take health care closer to the people. The corporatization trend is similar to what is happening in the retail segment; retail trade has traditionally been highly fragmented and predominantly ‘unbranded,’ but that is changing all over the country. In a similar way, the health care delivery system in India is still largely fragmented and over 95% of facilities are ‘one establishment’ entities. This is true for hospitals, stand-alone diagnostic centers, etc. However, the last 4-5 years have seen the beginning of corporatization and consolidation and this trend is going to accelerate further. In the next 10 years, in my estimate, close to 50% of the diagnostic centers will be part of a national or a regional chain. This could radically change the radiologist's perspective of his or her career and his or her potential to be an entrepreneur. The other related development is the expected huge capacity addition in the ‘hospitals’ sector. As per an FICCI study, India needs to add 800,000 beds in the next 10 years. This translates into 350 new hospitals (of 200-bed capacity) to be built every year for the next 10 years!! Most of these new hospitals will be created in tier II and tier III cities and towns. Hospital-based radiology practice will bring with itself the added aspect of joint patient evaluation by the radiologist and the referring physician. As of today, most of the radiology imaging is done in stand-alone diagnostic centers as opposed to hospitals and, in many cases, radiologists do not evaluate patients clinically. Often, they do not even see the patients, as they restrict themselves to viewing the images and reporting. In such a system there is also very poor follow-up of a patient's progress.

As a result of this, the radiologist is currently often not seen as a clinical/solution partner of the treating doctor. In the future, as more and more radiology imaging will happen in the hospitals, radiologists will be closely evaluating the patient before imaging is done and will also have an opportunity/protocol to follow the short-term and long-term progress of the patient. This will increase complexity at the workplace, while at the same time enhancing radiologists' job satisfaction. This could also lead a surge in the scientific temper in the health care community, with the radiologist playing a crucial part. Currently, the number of research projects being pursued and the amount of scientific papers published by the Indian health care community is woefully low. This is because, in many hospitals, there is a lack of communication between the various participants, namely, the referring doctor, the pathology service, and the imaging department. Usually they work in isolation, the imaging (radiology) often being done outside the hospitals. There is a good possibility that this will change.

Even if only one-third of this potential materializes in reality, this will change the employment/market potential for radiologists. In short, more and more radiologists will be working for an organization, rather than as entrepreneurs and this will have some positive impact. Radiologists will no longer have to bother about economic viability and the administrative needs of the organization, which will give them more time to focus on the clinical aspects. The reduced opportunities to start one's own enterprise could also have an impact on how much students are willing to spend to learn radiology. I was shocked when I heard a few years back that in some institutes, students are paying close to 10 million rupees to study/qualify as radiologists. In future, we might see this change drastically.

## Technological Trends

The pace of innovation in the diagnostics industry will only accelerate further. The gadgets will get smarter, faster, and more patient-friendly. There are several examples of these. The developments in CT scans are a good example. The industry has seen the introduction of 64-slice CT scanners and dual-source CT scanners; now 256- and 320-slice CT scanners are knocking at the door. On the MRI front, 3.0-T is slowly becoming the norm. Conventional x-ray based radiography is turning fully digital. Globally, 7.0 and 9.0 Tesla-based MRIs are now beginning to be used for human trials. Interventional radiologists have seen hybrid-based technologies become a reality. Cathlabs are doubling up as CT scanners as well. Intraoperative MRIs are beginning to show up. PET/CTs have blurred the boundary between radiology and nuclear medicine. Besides the evolution of the technological platform, there have also been rapid advances in the development of software. Computer-aided diagnosis (CAD) is now beginning to make its impact. In lung nodule detection and in mammography, CAD solutions are a clear reality. As these solutions are very well accepted by the industry, we will see further significant developments of a similar nature in many technologies, including CT scan, USG, and MRI.

All this implies that the radiologist has to keep pace with these developments in technology. My own personal experience of the last 10 years in the industry has shown me that radiologists are not investing enough time to understand/update their knowledge about even the basic issues in newer technologies. As a result, they tend to get easily carried away by different arguments and seldom are they able to personally evaluate different technology providers. In many instances, I have found radiologists struggling to calculate the x-ray dose delivered, for example, by a CT scanner. It appears to me that many of them are not taking such topics seriously enough. Understanding ‘temporal resolution’ in a cardiac CT scanner is another classical example where, if you talk to five different radiologists, you are likely to get six different definitions. The continuing development of new technologies will make it tougher for many radiologists to understand and correctly deploy these technologies. To start with, radiologists will need to understand the basic physics of these technologies as well as be very comfortable in dealing with sophisticated computerized solutions.

Besides the advances in imaging equipment and technology, developments in networking and bandwidths can be expected to have a huge impact. Data transfer speeds are going up from Kbps to Mbps and, eventually, to Gbps. This will make teleradiology and telemedicine more commonplace than is the case today. Teleradiology will spur investments in high-end diagnostics in smaller towns, as today the nonavailability of good-quality radiologists is seen as a major hindrance. While teleradiology within the country will become commonplace, the potential of cross-country and cross-continental teleradiology is also likely to be realized. With bandwidth prices crashing, the investments needed to set up a telereporting facility will go down significantly and that will encourage even more players to get into this space.

## Patient Awareness and Regulatory/Quality Trends

Patients are becoming more and more knowledgeable about health care. In many instances, when a patient is advised to get some form of imaging done, he/she does a search on the internet and tries to understand as much as possible about the clinical need, expected outcomes, etc. In the future, more and more patients will be asking radiologists or referring doctors why a particular technology has been chosen or why something has *not* been chosen. Radiologists should be mentally prepared to ‘discuss’ such issues with the patients and must learn to give satisfactory responses. There will be more pressure on radiologists to stay current with their knowledge. At present, there are no regulations in India that compel radiologists to regularly clear some tests/examinations or accrue minimum continuing medical education (CME) credits. This is one of the reasons why many radiologists are not exerting themselves to keep abreast of the advances in their field. In my estimate, within the next 3-5 years, our country will have in place clear guidelines and even the concept of ‘licensing’ and regular renewal of licenses. This will be another positive trend, which will further the cause of science and also ensure that the patient is benefited. More and more hospitals and diagnostic centers will work to get domestic/international accreditations and this will bring into greater focus the various processes covering imaging protocols, documentation of reports, etc. Currently, radiology is a highly ‘unregulated’ practice and this will change quite significantly within the next 3-5 years.

Overall, from a macro viewpoint, the Indian health care industry will continue to grow healthily at over 15% per annum for the next 5-10 years and this will keep the demand for radiologists quite high. Besides the sheer growth in volume, the advances in infrastructure and technology, and the changes in areas such as regulatory requirements, quality assurance, and patient awareness will make the practice of radiology even more exciting. At the same time, it will make many demands of the radiologist; for example, the radiologist may have to forgo autonomy to be part of larger organizations, get involved in patient follow-ups, and keep pace with technological jumps. In short, the future is both exciting and challenging for the practice of radiology in India.

